# Factors that influence biological survival in rheumatoid arthritis: results of a real-world academic cohort from the Netherlands

**DOI:** 10.1007/s10067-020-05567-6

**Published:** 2021-01-07

**Authors:** Elise van Mulligen, Saad Ahmed, Angelique E. A. M. Weel, Johanna M. W. Hazes, Annette H. M. van der Helm- van Mil, Pascal H. P. de Jong

**Affiliations:** 1 Department of Rheumatology, Erasmus MC, Room Na-523, Postbus 2040, 3000 CA Rotterdam, the Netherlands; 2 Department of Rheumatology, Maasstad Hospital, Rotterdam, the Netherlands; 3 Erasmus School of Health Policy & Management, Rotterdam, the Netherlands; 4 Department of Rheumatology, LUMC, Leiden, the Netherlands

**Keywords:** bDMARDs, Biological survival, Rheumatoid arthritis, TNF inhibitor

## Abstract

We aim to explore real-world biological survival stratified for discontinuation reason and determine its influenceability in rheumatoid arthritis (RA) patients. Data from the local pharmacy database and patient records of a university hospital in the Netherlands were used. RA patients who started a biological between 2000 and 2020 were included. Data on age, anti-citrullinated protein antibody (ACPA) and rheumatoid factor (RF) status, presence of erosions, gender, body mass index, time to first biological, biological survival time, use of csDMARDs, and discontinuation reasons were collected. Of the included 318 patients, 12% started their first biological within 6 months after diagnosis. The median time to first biological was 3.6 years (95% CI, 1.0–7.2). The median survival of the first- and second-line biological was respectively 1.7 years (95% CI, 1.3–2.2) and 0.8 years (95% CI, 0.5–1.0) (*p* = 0.0001). Discontinuation reasons for the first-line biological were ineffectiveness (47%), adverse events (17%), remission (16%), pregnancy (30%), or patient preference (10%). Multivariable Cox regression analyses for discontinuation due to inefficacy or adverse events showed that concomitant use of csDMARDs (HR = 1.32, *p* < 0.001) positively while RF positivity negatively (HR = 0.82, *p* = 0.03) influenced biological survival. ACPA positivity was associated with the inability to discontinue biologicals after achieving remission (HR = 1.43, *p* = 0.023). Second-line TNF inhibitor survival was similar between patients with a primary and secondary non-response on the first-line TNF inhibitor (HR = 1.28, *p* = 0.34). Biological survival diminishes with the number of biologicals used. Biological survival is prolonged if patients use csDMARDs. RF was negatively associated with biological survival. ACPA was negatively associated with the inability to discontinue biologicals after achieving remission. Therefore, tailoring treatment based upon autoantibody status might be the first step towards personalized medicine in RA.

**Table:** 

**Key Points** • *Prolonged biological survival is a surrogate for treatment effectiveness; however, an increasing amount of patients will taper treatment due to remission, and factors influencing biological survival based on separate reasons for discontinuation have not been explored*. • *We found that combining a biological DMARD with a conventional synthetic DMARD increases biological DMARD survival. Rheumatoid factor is negatively associated with biological survival. Anti-citrullinated protein antibody is negatively associated with the inability to discontinue the biological when remission was reached.* • *The first step towards personalized medicine might be tailoring of treatment based upon autoantibody status.*

**Supplementary Information:**

The online version contains supplementary material available at 10.1007/s10067-020-05567-6.

## Introduction

Management of RA has improved in the last decades due to early diagnosis, a treat-to-target approach, and the introduction of biological disease-modifying anti-rheumatic drugs (bDMARDs) [1]. Tumor necrosis factor inhibitors (TNF inhibitors) were the first bDMARDs to be developed for rheumatic diseases and are currently most frequently prescribed after an inadequate response to conventional synthetic (cs)DMARDs. It has been suggested that prolonged biological survival is a surrogate for treatment effectiveness [2]. Since more patients reach remission nowadays, more patients will be able to taper and discontinue treatment [1]. Therefore, solely taking into account overall biological survival will dilute outcomes, and to properly analyze biological survival, results should be stratified according to discontinuation reasons.

Previous studies, based on biological registries throughout Europe, have shown that 50% of patients discontinue their TNF inhibitor within 3–5 years [3]. Main reasons for discontinuation were inefficacy and adverse events [3, 4]. Within trials and biological registries, longer survival times were seen for first-line biologicals and when bDMARDS were combined with csDMARDs [5–7]. However, factors influencing biological survival based on separate reasons for discontinuation have not been previously explored.

Therefore, the aim of this Dutch real-world rheumatoid arthritis cohort is to explore first- and second-line biological survival and to determine its influenceability when stratified for discontinuation reasons.

## Patients and methods

### Study design

Data from a retrospective cohort were used, which we derived from the local pharmacy database and patient records of the Erasmus MC, an academic hospital in the Netherlands. We included data from rheumatoid arthritis (RA) patients starting a biological between 2000 and 2020. We excluded patients for whom non-adherence was reported, and if start and stop dates for bDMARDs were not available. Standard treatment of RA in the Netherlands is based upon a treat-to-target approach aiming for low disease activity. Methotrexate, unless contra-indicated, is the first choice of treatment after being diagnosed with RA. If the treatment target is not reached, another conventional synthetic (cs)DMARD can be started. If patients have an inadequate response to > 1 csDMARD, a bDMARD can be prescribed. In case of an inadequate response, rheumatologists can prescribe another bDMARD with the same mode of action (cycling) or a bDMARD with another mode of action (switching) [8].

### Data collection

Biological survival was the main outcome. Discontinuation was defined as skipping ≥ 2 doses and/or ≥ 2 months without biological treatment. Reasons for discontinuations were evaluated and classified into inefficacy, which we divided into primary (< 6 months) and secondary (≥ 6 months) non-response; adverse events (AEs); remission; pregnancy; patient preference; and other reasons.

### Analyses

We compared first- and second-line biological survival with Kaplan-Meier curves and with Wilcoxon-Breslow-Gehan tests at 3 years. Thereafter, first-line biological survival with and without concomitant use of csDMARD(s) was compared. Subsequently, we investigated whether primary and secondary inefficacy to a first-line TNF inhibitor leads to differences in second-line TNF inhibitor survival. Patients stopping their bDMARD due to remission or pregnancy were censored.

Cox proportional hazard models were used to estimate hazard ratios (HRs) of candidate baseline predictors (age, gender, ACPA, RF, erosions, BMI, DAS28, disease duration, or co-medication) for bDMARD survival stratified for reasons for discontinuation, namely (1) inefficacy or adverse events and (2) remission. First univariable Cox regression analyses were performed, and candidate predictors with a *p* < 0.20 were entered into a multivariable model, after which backward selection was applied until significance was reached. To prevent overfitting, an entry model was created and backward selection was applied. Schoenfeld residuals were assessed to check the proportional hazard assumption.

All data was analyzed using STATA 15. *p* values ≤ 0.05 were considered statistically significant.

## Results

### Patients

Data were derived from 318 RA patients (Table 1). Time until first bDMARD prescription remained constant between 2000 and 2020. In our cohort, 50% of patients started their first biological after 2013; thus, in most recent years, more bDMARDs were prescribed. A total of 39 (12%) patients started their first bDMARD within 6 months after diagnosis.

**Table 1:** Characteristics of rheumatoid arthritis population using a biological in a university hospital

	RA patients, *n* = 318
Demographic	
• Age at diagnosis, mean (SD)	40.9 (16)
• Gender, female, *n* (%)	264 (83)
• BMI, mean (SD)	26.9 (6.3)
Disease characteristics	
• ACPA positive, *n* (%)	224 (70)
• RF positive, *n* (%)	226 (71)
• Erosive disease, *n* (%)	141 (44)
Medication	
• Time to first biological (years), median (IQR)	3.6 (1–7)
• First-line biologicals	
○ Etanercept, *n* (%)	142 (45)
○ Adalimumab, *n* (%)	90 (28)
○ Certolizumab pegol, *n* (%)	59 (19)
○ Infliximab, *n* (%)	15 (5)
○ Golimumab, *n* (%)	5 (2)
○ Anakinra, *n* (%)	3 (1)
• csDMARDs used with first-line biological	
○ MTX, *n* (%)	66 (21)
○ MTX + SASP and/or HCQ, *n* (%)	147 (46)
○ Other csDMARDs (SASP, HCQ, LEF), *n* (%)	53 (17)
○ No combination therapy, *n* (%)	52 (16)

*ACPA* anti-citrullinated protein antibody, *BMI* body mass index, *csDMARD* conventional synthetic disease-modifying anti-rheumatic drug, *HCQ* hydroxychloroquine, *IQR* interquartile range, *LEF* leflunomide, *MTX* methotrexate, *RF* rheumatoid factor, *SASP* sulfasalazine, *SD* standard deviation

### First- and second-line biological survival

The median (95% CI) survival time of the first-line biological was 1.7 years (1.3–2.2), and for the second-line bDMARD, 0.8 years (0.5–1). The most prescribed first-line bDMARDs were etanercept (45%), adalimumab (28%), and certolizumab pegol (19%) (Table 1). Since only 9% of patients were using non-TNF inhibitors as second-line bDMARD, a direct comparison between a cycling and switching strategy could not be performed.

**Fig. 1:**
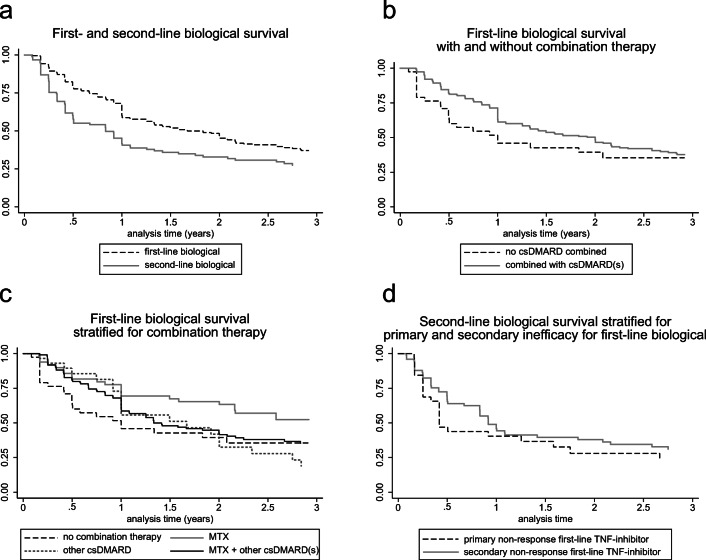
Kaplan-Meier curves for biological survival. **a** Kaplan-Meier for first- versus second-line biological survival. **b** Kaplan-Meier curve of patients with or without combination therapy. **c** Kaplan-Meier curve of patients without combination therapy, and for patients with combination therapy stratified for methotrexate, methotrexate combined with one or more other csDMARDs (sulfasalazine, hydroxychloroquine, and/or leflunomide), or one or more other csDMARDs. **d** Kaplan-Meier of second-line TNF inhibitor survival, stratified for primary and secondary inefficacy for the first-line TNF inhibitor. csDMARD, conventional synthetic disease-modifying anti-rheumatic drug; MTX, methotrexate

bDMARD survival was significantly longer for the first-line bDMARD compared to the second (*p* = 0.0001) (Fig. 1a). Discontinuation reasons for the first-line bDMARD were inefficacy (47%), adverse events (17%), remission (16%), pregnancy (30%), or patient preference (10%). Discontinuation reasons for the second-line bDMARD were similar (supplemental table S1).

### First-line biological survival with or without concomitant use of csDMARDs

A total of 48 (25.3%) and 6 (15.4%) patients respectively with and without concomitant use of csDMARD(s) were still using their first-line biological after 3 years of follow-up. The median (95% CI) survival time of the first-line bDMARD with csDMARD(s) was 2.0 (1.3–2.3) years, and without csDMARDs, 1.0 (0.5–5.3) year (Fig. 1b, *p* = 0.031). First-line bDMARD survival was longest for treatment regimens with methotrexate (MTX) followed by other csDMARDs, and no csDMARD use (Fig. 1c). However, no significant differences were found between MTX and the other csDMARDs as concomitant therapy (*p* = 0.14) (Fig. 1c).

### Primary and secondary failure

The median (95% CI) survival time for the second-line TNF inhibitor was 0.42 (0.25–1.58) years for patients with a primary non-response for the first TNF inhibitor and 0.92 (0.83–1.83) years for patients with a secondary non-response for the first TNF inhibitor. Although overall survival time on the second-line biological did not differ significantly between patients with a primary and secondary non-response (HR 1.28, *p* = 0.34), a trend could be observed (Fig. 1d).

### Predictors for biological survival

Univariate Cox regression for discontinuation due to inefficacy and adverse events showed that RF (HR = 0.80, *p* = 0.014) and presence of erosions (HR = 0.65, *p* < 0.001) were negatively associated with bDMARD survival. Concomitant use of csDMARD(s) (HR = 1.35, *p* < 0.001) on the other hand was positively associated with bDMARD survival. The aforementioned factors as well as time to bDMARD, age, gender, and ACPA were included in our multivariable model with backward selection. In the final model, only RF (HR = 0.82, *p* = 0.03) and concomitant use of csDMARDs (HR = 1.32, *p* = 0.001) were significantly associated with bDMARD survival (Table 2). When we used an entry model and applied backward selection, the aforementioned predictors were again in the final model, but also the presence of erosions was included.

**Table 2:** Predictors for overall biological survival

	Univariable		Multivariable^1^	
	HR (95% CI)	*p*	HR (95% CI)	*p*
Biological survival taking into account discontinuation due to inefficacy or AEs^2^				
Age at diagnosis	1.00 (1.00–1.01)	0.514		
Gender (female)	1.00 (0.82–1.23)	0.985		
BMI	0.99 (0.98–1.01)	0.296		
Rheumatoid factor	**0.80** **(0.67–0.96)**	**0.014**	**0.82 (0.69–0.98)**	**0.03**
ACPA	0.90 (0.75–1.07)	0.223		
Erosions	**0.65 (0.55–0.76)**	**< 0.001**		
Time to first-line biological	**0.98 (0.95–1.01)**	**0.163**		
Combination therapy	**1.35 (1.14–1.59)**	**< 0.001**	**1.32 (1.13–1.57)**	**0.001**
DAS28 at time of discontinuation	1.02 (0.87–1.21)	0.754		
Prolonged biological survival due to inability to taper^3^				
Age at diagnosis	1.00 (0.99–1.01)	0.717		
Gender (female)	1.08 (0.75–1.56)	0.676		
BMI	**0.98 (0.95–1.01)**	**0.175**		
Rheumatoid factor	**1.26 (0.94–1.96)**	**0.121**		
ACPA	**1.43 (1.05–1.93)**	**0.023**	**1.43 (1.05–1.93)**	**0.023**
Erosions	0.70 (0.53–0.92)	0.481		
Time to first-line biological	**1.04 (0.99–1.09)**	**0.119**		
Combination therapy	0.93 (0.69–1.26)	0.643		
DAS28 at time of discontinuation	0.81 (0.49–1.36)	0.430		

^1^Backward selection, variables with *p* < 0.20 in univariable analyses were entered. ^2^HR > 1 indicates prolonged biological survival; HR < 1 indicates reduced biological survival due to inefficacy or AEs. ^3^HR > 1 indicates prolonged biological survival due to inability to taper; HR < 1 indicates reduced biological survival due to tapering of bDMARD due to remission. Bold numbers within the univariable column indicate HRs which had a *p *< 0.2, bold numbers within the multivariable column indicate HRs with a *p* < 0.05

*ACPA* anti-citrullinated protein antibody, *AEs* adverse events, *BMI* body mass index, *CI* confidence interval, *DAS* Disease Activity Score, *HR* hazard ratio

The same procedure was followed for investigating which factors were associated with a higher chance of discontinuing bDMARDs due to remission. Only a positive ACPA status was associated with longer biological survival due to inability to taper medication (HR = 1.43, *p* = 0.023) (Table 2).

## Discussion

Optimal management of RA is based on reaching the lowest possible disease activity with a treat-to-target approach [1]. Despite the improved management approach and increasing treatment options, only 60–70% of RA patients will reach a long-term clinical response [4]. Within our study, we found a significant difference in survival time between the first- and second-line bDMARD, implicating the importance to prolong first-line bDMARD survival. Several factors can influence bDMARD survival of which some can be influenced.

Main reasons for discontinuation in our and in other studies were inefficacy and adverse events [3]. Primary inefficacy indicates no effect at all, and is thought to be due to a mismatch between the bDMARD and the specific RA subtype, causing the biologic agent not to be effective [9]. Secondary inefficacy indicates that the clinical response is first obtained, but not maintained, and is thought to be caused by formation of autoantibodies against the biologic [4]. Although we did not find a significant difference in second-line TNF inhibitor survival between RA patients with a primary or secondary non-response to the first TNF inhibitor, a trend could be observed. This was probably due to a low number of patients in the group with a primary non-response for the first-line bDMARD (*n* = 42). However, these data indicate that rheumatologists should consider switching to another mode of action in case of primary inefficacy instead of cycling to another TNF inhibitor, but validation is needed [10, 11].

Compared to previous findings, bDMARD survival seems to be short. This can be explained by the setting of our cohort in a tertiary care university hospital, which usually includes more difficult to treat and/or refractory RA patients. Furthermore, within an academic environment, patients are often participating in (treatment) trials, which could influence the results. For instance, the treatment in the Rotterdam Early Arthritis Cohort (tREACH) trial and the tapering strategies in RA (TARA) trial were initiated in the Erasmus MC [12, 13]. Furthermore, the Erasmus MC participated in the POET trial, which was also a tapering trial [14]. However, these trials were all following a treat-to-target strategy, which probably did not influence our results much. Besides these trials, a large proportion of the patients discontinued their bDMARD due to pregnancy. This is related to the fact that Erasmus MC has an ongoing cohort for patients with a wish to conceive [15]. Consequently, a high number of patients were using certolizumab pegol, which is known to be safe to use during pregnancy.

Outcomes of our study on the other hand are in accordance with previous findings. Benefits of combining a bDMARD with a csDMARD have been previously described [6, 16, 17]. Reasons for this synergistic effect are not fully understood. Soliman et al. investigated the csDMARDs separately and found the strongest effect on prolongation of biological survival when MTX was combined [16]. Unfortunately, we lacked power to confirm this for subgroups within the csDMARDs in our study. One of the reasons could be that csDMARDs can prevent development of neutralizing anti-drug antibodies. It is also thought that csDMARDs affect clearance of the bDMARD by modulating either the expression of Fc receptors on monocytes or the interaction of the Fc receptor and the bDMARD [4].

Another factor that could influence biological survival is the degree of adherence. The longer a patient has the disease, and uses a certain drug, the lower the adherence [18]. Furthermore, patients’ beliefs on the efficacy of the drug could play a role [19, 20]. For example, we already showed that biological survival improves if combined with a csDMARDs. And if patients believe that the csDMARDs are necessary, their compliance will increase, which will probably result in an even better biological survival.

Previous literature already showed that the presence of autoantibodies in RA is associated with a worse treatment response and outcome. Moreover, autoantibody-negative RA patients have a better treatment response compared to autoantibody-positive RA patients when given similar therapies [21]. In accordance with previous literature, we found a shortened biological survival due to inefficacy or adverse events in RF-positive RA patients but also the inability to taper TNF inhibitors after reaching remission in ACPA-positive RA patients [22]. This reconfirms the fact that autoantibody positivity is associated with worse outcomes and indicates that RA can be subdivided into autoantibody-positive and autoantibody-negative RA. This also suggests that treatment maybe stratified on autoantibody status, but validation is needed.

In conclusion, bDMARD survival diminishes with the number of bDMARDs used. Combining a bDMARD with a csDMARD increases bDMARD survival, which supports current EULAR recommendations to combine a bDMARD with a csDMARD. RF and ACPA were negatively associated with respectively bDMARD survival and discontinuation due to remission. Therefore, the possible first step to personalized medicine in RA might be tailoring of treatment based upon autoantibody status.

## Supplementary information

ESM 1 (DOCX 13 kb)

## Data Availability

Data are available upon reasonable request by contacting the corresponding author.
