# Supplementary material for: Factors that influence biological survival in rheumatoid arthritis: results of a real-world academic cohort from the Netherlands
Source: Clin Rheumatol. 2021 Jan 7;40(6):2177–83. doi: 10.1007/s10067-020-05567-6 (PMC8121743; doi:10.1007/s10067-020-05567-6)
Supplement: Supplementary file 1 — (DOCX 13 kb) [file 10067_2020_5567_MOESM1_ESM.docx]

**Table S1 Discontinuation reasons for first- and second-line biological**

|  | **First-line biological (n=318)** | **Second-line biological (n=192)** |
| --- | --- | --- |
| **Total number of patients discontinuing biological treatment** | 226 (71) | 127 (66) |
| **Ineffective** | 106 (47) | 63 (51) |
| **Adverse event** | 38 (17) | 28 (23) |
| **Remission** | 35 (16) | 13 (10) |
| **Pregnancy** | 30 (13) | 15 (12) |
| **Patient preference** | 10 (4) | 4 (3) |
| **Unknown** | 6 (3) | 1 (2) |
| **DAS28 at time of discontinuation, mean (sd)** | 3.19 (4.8) (n=107) | 2.90 (1.4) (n=52) |
| All results are indicated as n (%), unless indicated otherwise. DAS: disease activity score, sd: standard deviation | | |
